# Experts use base rates in real-world sequential decisions

**DOI:** 10.3758/s13423-021-02024-6

**Published:** 2021-10-26

**Authors:** Daniel Link, Markus Raab

**Affiliations:** 1 Department of Exercise Science and Sports Informatics, Technical University Munich, Georg-Brauchle-Ring 60/62, 80992 Munich, Germany; 2 Institute of Psychology, German Sport University Cologne, Am Sportpark Müngersdorf 6, 50933 Cologne, Germany; 3 School of Applied Sciences, London South Bank University, 103 Borough Road, London, SE1 0AA UK

**Keywords:** Base rate, Choice, Hot hand, Sport

## Abstract

Human behavior is often assumed to be irrational, full of errors, and affected by cognitive biases. One of these biases is base-rate neglect, which happens when the base rates of a specific category are not considered when making decisions. We argue here that while naïve subjects demonstrate base-rate neglect in laboratory conditions, experts tested in the real world do use base rates. Our explanation is that lab studies use single questions, whereas, in the real world, most decisions are sequential in nature, leading to a more realistic test of base-rate use. One decision that lends itself to testing base-rate use in real life occurs in beach volleyball—specifically, deciding to whom to serve to win the game. Analyzing the sequential choices in expert athletes in more than 1,300 games revealed that they were sensitive to base rates and adapted their decision strategies to the performance of the opponent. Our data describes a threshold at which players change their strategy and use base rates. We conclude that the debate over whether decision makers use base rates should be shifted to real-world tests, and the focus should be on when and how base rates are used.

It is a widely held belief that humans are irrational and show systematic violations of norms in their judgments (Nisbett & Ross, 1980; Pohl, 2016). One of the most debated biases is base-rate neglect, which refers to people not considering the base rates of a specific category when making decisions. Kahneman and Tversky’s (1973) famous example was a starting point for discussions of base-rate neglect. Participants in this study rated the probability that Jack, a person described as being—among other things—a conservative, careful, and ambitious 45-year-old man, was an engineer. Those ratings were much higher than the percentage of engineers in the presented sample. The authors’ argument, based on the representativeness hypothesis, was that people’s judgments reflect the essential features of the evidence—in this case, the person’s description rather than the base rates—leading to neglect of base rates. In contrast, Gigerenzer et al. (1988) found that 77% of participants actually did rely on base rates when estimating the probability of a team winning a game, a judgment identical in format to the engineer problem, and none reported using a representative heuristic. Such discrepant results have fueled the theoretical debate on whether base-rate neglect exists and highlight the need for the development of a prescriptive theory in realistic decision environments (Koehler, 1996).

How can these incongruent findings be resolved? Koehler (1996) argued that to overcome the inconsistencies, “patterns of base-rate usage must be examined in more realistic contexts to determine when, if ever, people make consequential errors” (p. 14). We argue here that to test this we need to go beyond lab experiments to solve the problems raised repeatedly by researchers in this field (Turpin et al., 2020). Therefore, the aim of the current study was to test whether base rates are used in real-world sequential decisions.

Many tasks people carry out in daily life are quite different from what has been tested in base-rate neglect research. A more appropriate test would therefore be one conducted under conditions that reflect a task–person match that can be observed in the real world. A recent review of probabilistic reasoning supports this idea (Schulze & Hertwig, 2021). This suggestion tracks back to an early goal of this kind of research—that is, to understand human behavior in uncertain environments (Peterson & Beach, 1967).

In the context of sports, it has been shown that fans, athletes, and coaches take previous performance (e.g., base rates and recent success) into consideration when betting, passing balls as a playmaker, or making strategic decisions (Bar-Eli & Raab, 2006). For instance, consider beach volleyball (two players against two players) and the simple choice between serving to opponent Player A or serving to Player B to gain an advantage. This decision potentially depends on multiple factors—one being the base rate of successful receptions of the serve. If Player A and Player B differ in their base rate of serve receptions—if, for instance, Player A’s successful reception of a serve is 4 of 10 and Player B’s is 6 of 10—ignoring base rates seems likely if both players are served to equally.

Beyond base rates, recent performance, such as the so-called hot hand, may explain serving behavior. Believing in the hot hand means believing that there is a higher probability a player will score again after two or three previous successes than after two or three misses. Base rates and the hot-hand belief have not been well explored in sequential decisions (but see Raab et al., 2012). Demonstrating how observable sequential decisions in the real world take base rates into account when they matter (or not) can provide information when humans make adaptive choices (Turpin et al., 2020).

The hot-hand belief in sports would appear to be a phenomenon well suited to the study of base-rate use, given that people may ignore base rates if they simply focus on the last couple of actions of the players. Base rates in sports are extensively described in sports statistics, such as the season performance, averages on game day, in a specific match, or in a set within a match. Base rates in sports are quite exact and have a typical structure of seasons, games, and sets that allow setting meaningful boundaries for analyses. Indeed, players may consider someone as “hot” even if the perceived sequences are random (see Gilovich et al., 1985, for the seminal study; Bar-Eli & Raab, 2006, for a review; and Avugos et al., 2013, for a meta-analysis). Even if sequential performances are partly random, recent evidence has shown that the hot hand exists if analyzed appropriately (Csapo et al., 2015; Miller & Sanjurjo, 2018).

For instance, multiple experiments have indicated that athletes are sensitive to base rates and the performance fluctuations of players. In a lab study of adaptive choice, Raab et al. (2012) analyzed whether using the base-rate performance of teammates was less important or could potentially be ignored when one of the players was hot. In the long run, playing to a currently hot player who hit only the last two or three attempts but had a lower base rate over the course of the game than to an alternative player was not adaptive. Raab et al. showed, however, when analyzing athletes’ performances from real games that hot players are those with a higher base rate, and thus participants in the experiment did act adaptively. Participants’ behavior in the experiments may have reflected their knowledge of the game as experts, suggesting that base rates are not ignored. Thus, experiments and sports statistics have shown both the use and neglect of base rates (Avugos et al., 2013; Bar-Eli & Raab, 2006; Cohen, 2020).

Against this background, this paper explores the real-world decision behavior of experts using beach volleyball as a test bed, taking advantage of several game characteristics: (i) The serve decisions are binary (serve to Player A or Player B); (ii) expertise is easily scaled by the rankings of players in competitions; (iii) the sequential nature of decisions and their consequences can be measured and directly observed by those making the decisions; and (iv) game statistics and modern scouting practices provide big data on real choices.

Taking a descriptive approach first, we ask how stable base rates and selection rates (extent of being selected by the serving team) are and how strongly they differ between players (Question 1). This part is a mandatory precondition for testing base-rate use since a sufficient variation of base rates is needed. To consider potential effects of sex on performance and decision behavior, our analysis differentiates between male and female players.

When base rates and selection rates are known, we address the question of whether the player selection (serve to Player A or Player B) is influenced by the base rates of the players’ performance (Question 2). We argue that player performance within meaningful boundaries such as a previous set—that is, the players’ base rates—may serve as information to be considered in the next set when choosing to whom to serve. Likewise, previous games or season averages may inform choices in the next game to come. We assume that professional players want to maximize their chance of winning and therefore will serve to the player with a lower probability of scoring. We explored this question for different time frames and asked whether players use opponents’ base rates with respect to their overall performance in recent years (long-term information) or in the current match/set or previous set (medium-term information).

Testing more than 1,300 matches on the expert level also allowed us to check base-rate sensitivity thresholds. We explored what base-rate differences within teams change the allocation strategy in serving. What pattern of results in the data set of beach volleyball would count as evidence of base-rate neglect? When we detect base-rate differences, and the serve of the opposing player is systematically not served to the player with the lower base rate, or serves are played equally to both players, this would be an indication of base-rate neglect; otherwise, it would be an indication of base-rate use.

Base-rate information might not be the only type of information that is used by players when deciding to whom to serve. As already mentioned, Raab et al. (2012) showed that playmaker decisions in volleyball are also based on short-term performance information. To test this, we asked whether beach volleyball players perceive and consider recent performance changes in the last few rallies (short-term information) for their serving decisions (Question 3). We expected that a “hot” player would be selected less often compared with a player who was not successful in the recent rallies. If the answers to these questions is affirmative and players are sensitive to opposing players’ performance, the question remains as to whether this selection strategy was useful in terms of scoring. To clarify this, we also tested whether using versus neglecting base rates in the short term influenced the chance of scoring.

Answering these questions should help determine whether base rates matter in the real world and by extension provide support for either models that claim experts neglect base rates or models that claim experts use base rates (Turpin et al., 2020). Additionally, this research provides an opportunity to go beyond that basic dichotomy and outline the parameters of when and to what extent base rates are used.

## Methods

### Sample

The sample comprised 1,347 matches (565 men, 782 women) in the Fédération Internationale de Volleyball (FIVB) World Series 2012–2018 and the 2012 and 2016 Olympic Games. All procedures in the study were performed in strict accordance with the Declaration of Helsinki as well as with the ethical standards of the local ethics committee.

### Variables

Beach volleyball matches usually follow a best-of-three format, with two sets each played to 21 points and the third, deciding set, if needed, to 15 points. There are two basic situations within a rally. In the *sideout* situation, Team A receives Team B’s serve, passes the ball, and tries to score with an attack. Team B tries to defend the attack to score itself (the defense situation). The winner of a rally scores and will be the next to serve, so that sideout and defense switch continuously between the teams (FIVB, 2017). To answer our research questions, we focused on the sideout situation only. We argue that defense differs clearly from the sideout situation in terms of the skills needed for success (Giatsis et al., 2015). Therefore, defense performance is a negligible factor with regard to the decision as to which player to select to receive the service.

For each sideout, two variables were collected: The *selected player* was the player who received the serve. In the case of an *ace*—no player touched the ball before it hit the ground—the nearest player in the hitting moment was chosen as the selected player. Rallies with serve errors were excluded from the sample. The *outcome* of a sideout was considered *successful* (a *hit*) if the attacker scored a direct point or the opposing team was not able to touch the ball more than once. Otherwise, the sideout was designated *unsuccessful* (a *miss*). A miss does not imply that the sideout team did not win the rally, since there is still the possibility of defending the counterattack and scoring. The performance variables selected are standard in scouting reports (Link & Wenninger, 2019).

From these performance variables, we derived the selection rate and the base rate. The selection rate is given by the number of selections of a player divided by the number of selections of the team. The base rate of a player is calculated by all their individual successful sideouts divided by the number of selections within a given time frame. Rallies in which the other player plays the ball over the net on the second contact or there is a setting error caused by the other player are excluded from the calculation of the base rate. For the base rate analysis, only data on players selected at least 10 times per match or five times per set were included.

All data were annotated by professional beach volleyball analysts using custom-made observation software for use with video recordings (Link, 2014). The data are part of a more detailed data set that was used to prepare Germany’s national teams for their competitions and has already been used in other publications (Link & Wenninger, 2019; Wenninger et al., 2020). Cohen’s kappa statistics show substantial to perfect agreement between two observers for the variables selected player and outcome based on a subset of 130 sideouts (κ = .94 to 1.0).

### Statistical analysis

To test whether athletes use base rates, we first need to describe base and selection rates and their stability (Question 1). We report base rates and selection rates for male and female players and their variation long term and on the match level in box plots. Long-term analysis covered all matches of a player in the data set and included only players with at least 10 matches available. To test whether athletes use base rates for selection (Question 2), we calculated the correlation (Pearson’s *r*) of base rates and selection rates on the long-term, match, and set level. In the Set+1 configuration, we correlated the base rate in set *n* with the selection rate in set *n*+1. To check players’ sensitivity to base rate thresholds, we grouped sets by the base rate difference between teammates and correlated base rates and selection rates within these sets. To analyze whether athletes use short-term performance of opposing players for selection (Question 3), we looked at series of consecutive selections of a player and asked whether the selection rate of this player differed after successful and unsuccessful streaks. To test whether such streaks influenced performance, we also looked at whether the hit rate after successful and unsuccessful streaks differed. For answering Questions 3, all matches were used and all players were treated as one group.

Rate comparisons are reported as the percentage difference (Δ) from the reference value. To test the significance of rate differences between men and women, we used two-sided*t* tests. To test the significance of rate differences between positive and negative streaks, we conducted χ^2^ tests. Cohen’s *d* and Cramer’s *V* are used to describe the effect sizes of significant differences. We verified the assumption of normality before using parametric statistical test procedures. The α level was set to .05. All statistical analyses were performed using R (Version 4.1).

## Results

### Question 1: How stable are base rates and selection rates, and how strongly do they vary between players?

**Fig. 1:**
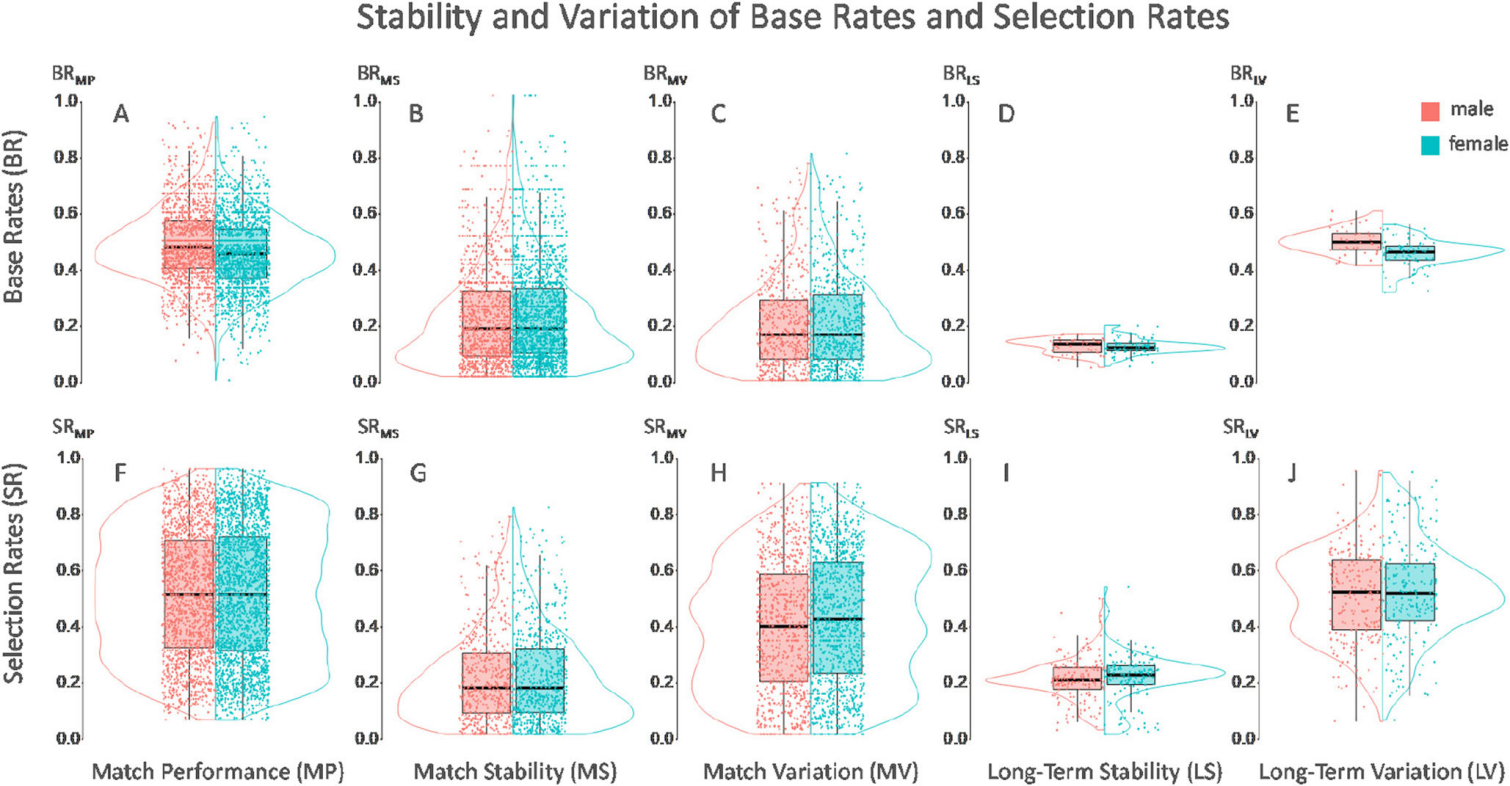
Stability and variation of base rates (BRs) and selection rates (SRs). The data show remarkable fluctuations in the base rates of one player from set to set (**b**), as well as between the players of one team (**c**). Both results argue in favor of players considering base rates when selecting a player when serving. Long-term variation (**e**) does not differ very much between players, which shows the homogeneity of the sample containing only the best athletes in the world in this sport. Players also showed a clear tendency to select one opponent more often than the other (**h**)

Figure 1 shows the range, stability, and variation of base rates and selection rates on different time scales. The analysis of *match performance*(MP) used Player × Match as the statistical unit (*n* = 2,975; Fig. 1a and Fig. 1f). Performance is reported as the mean base rate^1^ per match per player: BR_MP_ = .47 ± .13 (Fig. 1a). This reveals that success and failure in sideout were quite balanced. Analysis of performance stability of one player within one match (*match stability*, MS) used Player × Set Pair as the statistical unit (*n* = 4,072; Fig. 1b and Fig. 1g) and is reported as the magnitude of base rate difference (BR_MS_) from set *n* to set *n*+1 (Fig. 1b). To describe performance variation between the two teammates (*match variation*; MV), the analysis used Team × Match as the statistical unit (*n* = 1,898; Fig. 1 c and Fig. 1h). Differences between the two teammates were quantified by using the magnitude of their base rate difference (BR_MV_). Results show that base rates of one player differed between sets by 45.7% (BR_MS_ = .21 ± .17; Fig. 1b) and base rates between the players differed by 34.0% (BR_MV_ = .16 ± .13; Fig. 1c). Both results argue that players should consider base rates when selecting a player when serving.

Quantification of performance stability of one player between several matches (*long-term stability*, (LS; Fig. 1d and Fig. 1i) and long-term performance variation between players (*long-term variation*, LV; Fig. 1e and Fig. 1j) used player as the statistical unit (*n* = 120). Long-term variation was quantified as the mean base rate of players in all their matches in the entire sample and did not differ very much between players (BR_LV_ = .48 ± .05; see Fig. 1e). This is reasonable, since the sample represent a homogenous group containing only the best athletes in the world in this sport. In addition, the long-term stability, reported as the standard deviation of the base rate in all matches of one player, was lower compared with match stability (BR_LS_ = .12 ± .03; see Fig. 1d).

Selection rates were aggregated on the different time scales in the same way as shown for base rates. The match variation of selection rates revealed that players had a clear tendency to select one opponent more often than the other (SR_MV_ = .40 ± .23; see Fig. 1h). In 60.4% of the matches, one player was selected more than twice as often as their teammate. This leads to a higher variation of the selection rate between players compared with base rates and a higher distribution on match (see Fig. 1f) and long-term(Fig. 1j) levels.

Base rates of men were Δ = +3.0%, which is slightly higher compared with women on the match level (*t* = 6.1, *p* < .01, *d* = 0.23), and Δ = +4.3% higher on the long-term level (*t* = 4.9, *p* < .01, *d* = 0.89). Since there were no other effects regarding the factor sex, and the differences in base rates were quite small, we do not differentiate between men and women when studying Questions 2 and 3.

### Question 2: Is player selection influenced by the base rates of the players’ performance, and does a sensitivity threshold exist?

**Fig. 2:**
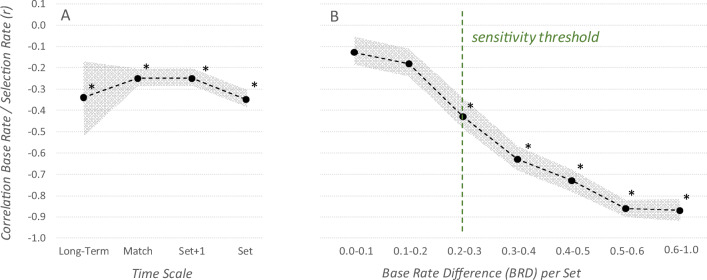
Use of base rates for player selection on long-term and mid-term level. Results show a negative correlation (r) between hit rates and selection rates (**a**). The stronger sideout player—in terms of the base rate—was selected less often and the weaker player was selected more often. **b** The correlation between hit rates and selection rates per set grouped by the base rate difference (BRD) of the teammates. The data imply a sensitivity threshold for use of base rates, which is located at a base BRD of about .25 (**b**). * indicates significant correlations, shaded band indicates 95% CI

Figure 2a shows the relationship between base rates and selection rates on the long-term, match, and set level. The long-term analysis used players as the statistical unit (*n* = 120), the match-level analysis used Player × Match as the statistical unit (*n* = 2,975), the Set+1 analysis used Player × Set Pairs as the statistical unit (*n* = 4,054), and the analysis on the set level used Player × Set as the statistical unit (*n* = 4,172).

There are three results resp. observations to be stressed. First, all conditions showed a negative correlation between the base rate and the selection rate. The stronger sideout player—in terms of the hit rate—was selected less often and the weaker player was selected more often. Second, the correlation of base rates in set *n* and base rates in set *n*+1 was *r* = .26, which is at a similar magnitude to its prediction of whether the player would be selected as the person to whom to serve in set *n*+1 (*r* = -.25, see Fig. 2a, category Set+1). Third, the correlation of selection rates in set *n* and selection rates in set *n*+1 was *r* = .50, which is larger compared with the correlation of base rates in set *n* and base rates in set *n*+1 (*r* = .26). In other words, the decision of to whom to serve was more influenced by the past decision than the past outcome—players had a tendency to stick to their strategy.

To check players’ sensitivity to base-rate thresholds, we grouped sets based on the base-rate difference between the two players of one team. Seven groups were created; their subscripts indicate the range of base-rate differences contained in this group (e.g., Set BRD_[0.0-0.1]_ ≙ 0.0 < BRD ≤ 0.1). The number of statistical units was between *n* = 784 (BRD_[0.0-0.1]_) and *n* = 103 (BRD_[0.6-1.0]_) for subgroups. Results show a monotonically strengthening negative correlation between base rates and selection rates (see Fig. 2b). With increasing base rate difference within a team, the serving players selected the stronger player less often. The correlation is significant starting from group BRD_[0.2-0.3]_, which can be interpreted as the sensitivity threshold.

### Question 3: Is player selection influenced by short-term performance, and is this useful in terms of winning the next rally?

**Fig. 3:**
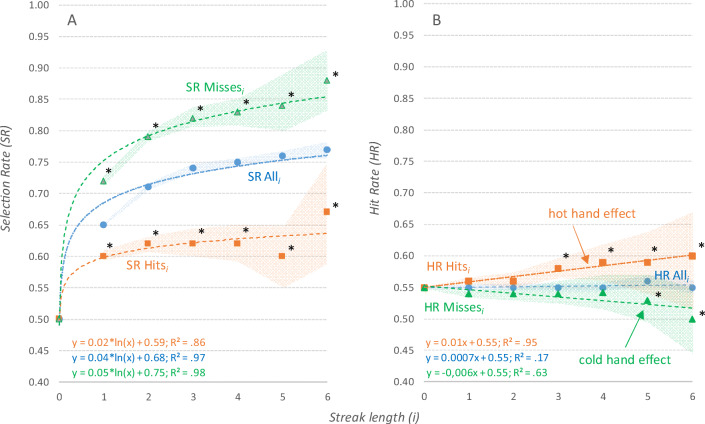
Influence of short-term performance on player selection (**a**) and subsequent player performance (**b**). **a** Selection rate (SR) after a sequence of hits (SR Hits_i_) were significantly lower compared with SRs after a sequence of misses (SR Misses_i_) as well as compared with the group of sequences containing all sideouts (SR All_i_). Values were interpolated by using logarithmic regression functions. **b** After a sequence of hits, the HR (HR Hits_i_) was higher compared with the hit rate after sequences containing all sideouts (HR All_i_). In the same way, HR Misses_i_ was smaller compared with HR All_i_. This effect increased with the length of the sequence and became significant after three hits in a row (hot hand effect) and after five misses in a row (cold-hand effect). Performance streaks influenced player selection rate much more than they influenced subsequent hit rates. Values were interpolated by using linear regression functions. * indicates significant differences compared with SR All (**a**) and HR All (**Part B**), shaded band indicates 95% CI.

Figure 3a shows the use of base rates for selection on a short timescale. It reports the selection rate after a sequence of *i* ∈ {1,…,6} consecutive selections of the same player in the same set. SR All_*i*_ represents the selection rate after *i* sideouts in a row and is calculated as the probability of this player being selected once again in the sideout *i*+1. SR Hits_*i*_ represents the selection rate after a series of *i* successful sideouts, and SR Misses_*i*_ represents the selection rate after a series of *i* unsuccessful sideouts. The analysis was based on a sample of *n* = 58,003 sideouts (All_0_). The size of the smallest subgroup was *n* = 141 (Hits_6_).

Results indicate that the selection rate tended to increase with the sequence length; with each selection, it became more likely that this player would be selected once again. Further, the selection rate was affected by the base rate in the previous sideout sequence. The selection rate after sequences of misses (SR Misses_*i*_)—in which BR = 0 by definition—was significantly higher compared with the selection rate after sequences containing all sideouts (SR All_*i*_)—in which BR = .55 (see Fig. 4)—for all *i*. In the same way, the selection rate after sequences of hits (SR Misses_*i*_)—in which BR = 1 by definition—was lower compared with SR All_*i*_ for all *i*. For example, the selection rate after two misses was SR Misses_2_ = .82 and the selection rate after two hits was SR Hits_2_ = .62, which is a significant difference of Δ = +27.1% (χ^2^ = 616.9, *p* < .001, *v* = .18).

Figure 3b shows the performance (hit rate) of players after successfully scoring *i* times in a row. The hit rate represents the probability of this player scoring in the next rally when being selected once again in the sideout *i*+1. HR All_*i*_ refers to the hit rates in the sideouts after sequences of length *i*. HR Hits_*i*_ and HR Misses_*i*_ represent the hit rates in the sideouts after a sequence of *i* hits and misses in a row, respectively. The analysis was based on a sample of *n* = 37,947 sideouts (All_0_). The size of the smallest subgroup was *n* = 98 (Hits_6_).

The data reveal that hit rates after sequences of misses (HR Misses_*i*_) were lower compared with hit rates after sequences containing all sideouts (HR All_*i*_; the cold hand effect). In addition, hit rates after sequences of hits (HR Hits_*i*_) were higher compared with HR All_*i*_ (the hot hand effect). After a sequence of six hits, for example, the hit rate was HR Hits_6_ = .60, and after a sequence of six misses, the hit rate was HR Miss_6_ = .50. From this perspective, selecting the player with a negative streak was more promising than selecting the player with the positive streak. With increasing sequence length, differences between bases rates tended to become larger. Significant differences compared with HR All_*i*_ occur from sequence length *i* = 3 for positive streaks (Δ = +4.8%, χ^2^ = 5.41, *p* < .05, *v* = .02) and from sequence length *i* = 5 for negative streaks (Δ = +2.9%, χ^2^ = 4.92, *p* < .05, *v* = .02).

## Discussion

Decisions on to whom to serve the ball in beach volleyball were used as a test bed to investigate the (ir)rationality of expert behavior. We asked whether experts used base rates as well as recent information in real-world decisions. The short answer is yes. We found that base rates and selection rates varied (Question 1) and that selection was based partly on base rates (Question 2) and short-term performance streaks and was functional in winning games (Question 3). A new finding is that even in complex real-life environments it is possible to analyze biases and test whether those biases are meaningful: Beach volleyball and most sports in general are environments in which feedback on choices is omnipresent. Environments such as sports in which decisions and outcomes are closely linked in time are called *kind* environments (in contrast to wicked environments; Hogarth et al., 2015) and are well suited for analyzing experts’ use of base rates.

The data show that the more the base rates deviated between the two players, the more a serving player used base rates to serve the ball to the opposing player with the lower base rate. Thus, the opposing player with the lower base rate was selected more often than base-rate neglect would predict. It seems that sequential information about the previous success of the opposing players produced a choice strategy of relying on the base rate differences of players (at least if the base rate difference was above a sensitivity threshold). Whereas the literature discussed in the introduction often asked single questions and provided percentage information, in the real world, information often needs to be sampled in sequences. Decisions are therefore more experienced than described, a classic distinction in decision making on how recent and temporally distant information is used (Hertwig et al., 2004). Even though in labs multiple trials have been used in choice tasks, the relation between sequential performance and base rates has not been analyzed. Our study indicates that the magnitude of the difference between base rates of two players systematically explains choices. This in itself adds to the literature because base rates are neither used nor neglected; rather, when a threshold of a base rate difference is met, experts change selection strategies. A selection strategy as to whom to serve in beach volleyball provides a detailed look into real-world choices. Understanding the functional value of base rate use and strategies relying on base rates may decide games. The findings stress the theoretical need to understand base rates and their use in dynamic and changing environments.

Our data show that scoring probabilities are affected after a series of at least three hits or five misses in a row (but not before). This finding indicates that (i) using short-term base rates for player selection is functional and helps win matches and that (ii) a minimum streak length is needed before hot hand or cold hand effects come into play. The different thresholds for positive and negative streaks might be interpreted to mean that beach volleyball players become more “hot” and less “cold.” Traditionally, hot hand research has defined a performance streak as a sequence of three or more consecutive hits or misses (Gilovich et al., 1985). It has often been argued that the number “three” is important, since the third repetition of an event leads to an impression of nonrandomness in an observer’s mind (Carlson & Shu, 2007). The results of our study suggest that further research is needed to show streak length and its interpretation may differ if sequential performance is observed or produced.

The current data provide evidence for the weighting of information on different timescales. Immediate runs of two have barely any significance in terms of opponent hit rates but influence selection rates by over 20%. On the other hand, medium-term performance predicts subsequent player selection with a similar magnitude to the subsequent performance. This suggests that short-term information disproportionally affects a player’s serving decision, whereas weighting of medium-term performance is proportional. This does not mean that long-term and medium-term base rates are not used in the decision, but player weight old and recent information differently.

As no study in the real world can control for all influencing factors, we cannot be sure that players perceiving base rates and using them for their choices was directly tested. The decision as to which players to select is driven not only by their base rates but also by additional strategies: Players may serve to the supposedly stronger player once to surprise them or select an opponent many times in a row to induce fatigue. The positive correlation of selection rate and sequence length in our data provides evidence reflecting the second strategy.

We argue that instead of resolving an old debate on *whether* there is a use or neglect of base rates, a new question of *when* base rates are used needs empirical evidence (Turpin et al., 2020). Appropriate base rate use is therefore an adaptation to the task of experts and generalizable to the principles of experts’ intuition (Kahneman & Klein, 2009). In a nutshell, accepting base-rate neglect as a universal bias in lab testing may itself produce biases, which we tried to overcome with big data from the real world. Future research might thus take advantage of combined research in lab and real-world conditions on when and how base rates are used.

## Conclusions

Analysis of the serving strategies in expert athletes in more than 1,300 beach volleyball games revealed that these experts are sensitive to base rates and adapt their decision strategies to the performance of their opponents. Our data show the existence of a threshold of base-rate differences (about 25%) at which players change their serving strategy and use base rates. The distribution of selection rates also suggests that players use a probability-matching strategy instead of selecting the weaker player in every trial. The findings provide supporting evidence for hot hand and cold hand phenomena in sequential decisions in real-world environments. We conclude that the debate as to whether base-rate neglect exists or not needs to be moved to real-world tests and should seek to specify when and how base rates are used.

## Footnotes

1 In the Results section, some key terms are abbreviated in the reported statistics: BR = base rate; BRD = base rate difference; HR = hit rate; SR = selection rate.
